# Working Memory in Aphasia: The Role of Temporal Information Processing

**DOI:** 10.3389/fnhum.2020.589802

**Published:** 2020-12-23

**Authors:** Mateusz Choinski, Elzbieta Szelag, Tomasz Wolak, Aneta Szymaszek

**Affiliations:** ^1^Laboratory of Neuropsychology, Nencki Institute of Experimental Biology of Polish Academy of Sciences, Warsaw, Poland; ^2^Bioimaging Research Center, World Hearing Center, Institute of Physiology and Pathology of Hearing, Kajetany, Poland

**Keywords:** aphasia, working memory, auditory speech comprehension, temporal information processing, time perception

## Abstract

Aphasia is an acquired impairment of language functions resulting from a brain lesion. It is usually accompanied by deficits in non-linguistic cognitive processes. This study aimed to investigate in patients with aphasia the complex interrelationships between selected cognitive functions: auditory speech comprehension, working memory (WM), and temporal information processing (TIP) in the millisecond time range. Thirty right-handed subjects (20 males) aged from 27 to 82 years suffering from post-stroke aphasia participated in the study. Verbal working memory (VWM) and spatial working memory (SWM) were assessed with: (1) a receptive verbal test and (2) the Corsi Block-Tapping Test, respectively. Both these WM tests used the forward tasks (mainly engaging maintenance processes, i.e., storing, monitoring, and matching information) and backward tasks (engaging both maintenance and manipulation processes, i.e., reordering and updating information). Auditory comprehension was assessed by receptive language tests, and TIP efficiency was assessed by auditory perception of temporal order in the millisecond time range. We observed better performance of forward WM tasks than backward ones, independently of the type of material used. Furthermore, the severity of auditory comprehension impairment correlated with the efficiency on both forward and backward VWM tasks and the backward SWM task. Further analysis revealed that TIP plays a crucial role only in the latter task. These results indicate the divergent pattern of interactions between WM and TIP depending on the type of WM tasks. Level of verbal competency appeared to play an important role in both VWM tasks, whereas TIP (which is associated with manipulation processes) appeared to be important for SWM, but only on the backward task.

## Introduction

Over the years, researchers focused on the distortion of verbal communication as the core symptom of post-stroke aphasia. However, it has become evident that people with aphasia (PWA) also display impairments in cognitive domains other than language functions, such as: executive functions (Purdy, 2002; Fridriksson et al., 2006), attention Murray, 2002, 2012; Hula and McNeil, 2008; Villard and Kiran, 2017), learning and memory (including working memory; Wright and Shisler, 2005; Mayer and Murray, 2012), as well as temporal information processing (TIP) considered to be a neural frame for many cognitive functions (Szelag et al., 2015). These non-language deficits may intensify communication difficulties and hinder the rehabilitation process (El Hachioui et al., 2014; Simic et al., 2019).

Despite the widespread awareness of deficiencies in language and other cognitive functions in PWA, the interactions of these functions have rarely been studied (Sung et al., 2009; Laures-Gore et al., 2011; Oron et al., 2015). Such relationships seem important as these functions interact in our working brain. The current study concerns complex interactions in PWA between selected cognitive functions; i.e., auditory speech comprehension, working memory (WM), and TIP (on the millisecond level). We expected that the deficient TIP observed in PWA would play a crucial role in both language and WM deficits.

### Multicomponent Model of WM

Working memory (WM) has been conceptualized as a limited capacity system, designed to maintain, process, and manipulate information over short periods of time (Baddeley, 2000, 2003). In an influential model developed by Baddeley and Hitch (Baddeley and Hitch, 1974; Baddeley, 2000), a system named central executive supervises three components: (1) the phonological loop which directs the rehearsal and maintenance of verbal information; (2) the visuo-spatial sketchpad which stores visual and spatial information; and (3) the episodic buffer which integrates information from these two subsystems and refers it to long-term memory.

Measuring memory span is one method commonly used to assess WM capacity. This paradigm includes a series of items presented to a subject, whose task is to reproduce the sequence either in the same order (forward task) or in the reverse one (backward task). While the forward task requires the storage and maintenance of information, the backward one, in addition to maintenance, requires active manipulation of memory traces.

In verbal working memory (VWM) tests, backward tasks are usually more difficult than forward ones, both in healthy and clinical populations (Kessels et al., 2008; Laures-Gore et al., 2011). While forward tasks depend mainly on the phonological loop, the manipulation necessary to reverse the order in backward tasks requires the engagement of the central executive. On the other hand, in spatial working memory (SWM) tests, like the Corsi Block-Tapping Test, the difference in performance between forward and backward tasks is less pronounced. For example, Kessels et al. (2008) reported that both tasks are equally difficult for healthy elderly people. Thus, it is hypothesized that both forward and backward SWM tasks rely mostly on the visuo-spatial sketchpad, as the spans need to be maintained as visual patterns, with no need for serial ordering (Smyth and Scholey, 1992). On the other hand, Vandierendonck et al. (2004) argued that both SWM tasks involve the central executive, especially for longer sequences, which may lead to similar difficulty levels on both tasks.

### Working Memory in PWA

For many years, WM has been extensively studied in PWA (De Renzi and Nichelli, 1975; Caspari et al., 1998). Researchers suspected that language impairments may result in deficient VWM because of language problems. But WM deficits have been observed not only in verbal, but also in visuo-spatial tests (Potagas et al., 2011), which suggests more general WM impairment in PWA (see above the multicomponent model of WM).

Potagas et al. (2011) showed significant correlations between WM performance and severity of aphasia. While associations between verbal spans and aphasia severity are well-documented, studies concerning visuo-spatial span give inconsistent results. While in the aforementioned study by Potagas et al. (2011), significant correlations were observed between aphasia severity and forward and backward SWM (Corsi Block-Tapping Test), their magnitudes were rather low (0.36 and 0.33 for these two tasks, respectively). On the other hand, Paulraj et al. (2018) did not observe any significant correlations between either forward or backward spatial spans and language functions (comprehension, repetition, fluency, or overall language score).

### Neuroanatomical Evidence for the Co-existence of WM and Language Deficits

Further support for the co-existence of WM and language deficits in PWA comes from neuroanatomical data. Using voxel-based morphometry, Leff et al. (2009) reported that the density of the posterior region of the left superior temporal gyrus predicted the efficiency of both auditory VWM capacity and comprehension of spoken sentences.

Moreover, most lesion studies reported that PWA displayed deficient WM, assessed by behavioral tests. For example, some studies (Baldo and Dronkers, 2006; Kasselimis et al., 2018) reported that PWA with lesions in the left inferior parietal cortex presented significant impairment in VWM (forward task). Moreover, Kasselimis et al. (2018) found decreased VWM performance (forward Digit Span) in PWA with lesions in the inferior frontal gyrus. Nevertheless, the authors did not identify the regions specifically associated with performance on either backward VWM tasks or SWM (both tasks).

Although, the execution of visuo-spatial tasks is traditionally associated with the right hemisphere (De Renzi et al., 1977), the deficient SWM in PWA suggests that the left hemisphere contributes to the processing of visuo-spatial stimuli (Martin and Ayala, 2004; Potagas et al., 2011). This has been confirmed by Paulraj et al. (2018) who showed that performance of backward SWM was poorer in patients with lesions in the left fronto-parietal network, including the somatosensory cortex, supramarginal gyrus, lateral prefrontal cortex, and frontal eye fields.

Based on results observed on both VWM and SWM tasks, Baldo and Dronkers (2006) concluded that the left parietal cortex is specialized for processing successive stimuli, regardless of the material presented.

To summarize, the neuroanatomical data cited above indicates that the left hemisphere also plays a role in the co-existence of deficits in language and WM (both verbal or spatial material).

### Temporal Information Processing in PWA

A number of studies have demonstrated a link between TIP and language. Human speech is constrained by temporal organization on different time levels. Millisecond TIP is related to phonological encoding/decoding and syllabification, while multisecond TIP is involved in lexical selection, sentence production, and perception (Szelag et al., 2015). The current study concerned associations between millisecond TIP, auditory speech comprehension, and WM.

Numerous studies have reported that millisecond TIP is involved in phonemic hearing, i.e., the ability to analyse and synthesize speech sounds. For example, information about the formant transition related to the place of articulation in stop-consonants is constrained on a time range of about 20–40 ms intervals (Szelag et al., 2014). Thus, the differentiation between stop consonants requires highly precise millisecond TIP. Accordingly, the phoneme identification impairment observed in PWA may be accompanied by deficient processing of rapid auditory stimuli. Deficient TIP is evidenced in PWA with lesions in left temporo-parietal cortices (von Steinbüchel, 1998; Wittmann et al., 2004). This corresponds to difficulties identifying phonemes, resulting in an inability to decode verbal auditory input. The severity of TIP deficits in the millisecond range is associated with deficient comprehension in PWA (Swisher and Hirsh, 1972; Tallal and Newcombe, 1978; Fink et al., 2006; Oron et al., 2015). Some recent studies have also confirmed significant correlations between TIP and phoneme discrimination on the level of words and sentences (Fink et al., 2006; Oron et al., 2015).

According to previous studies, TIP may be considered as a neural frame for many cognitive functions, including WM (von Steinbüchel and Pöppel, 1993; Szelag et al., 2011; Bao et al., 2013). As WM is characterized by temporal dynamics in the millisecond range, the distortion of the internal clock may severely impair the proper execution of mental functions (Nowak et al., 2016). It might be expected that both VWM and SWM tests require effective TIP — the verbal task due to linguistic processing and the SWM task due to the manipulation of processed material (which seems to be engaged mostly in the backward task). Such manipulation requires dynamic mental processes in which the temporal frame seems crucial.

On this basis, one may expect that TIP distortions caused by brain injury will be strongly associated with impairment of both auditory speech comprehension and WM. Thus, in the present study we explore the complex associations between WM, speech comprehension, and TIP.

### Experimental Aim

In the current study we compared the performance of PWA on VWM and SWM tests, considering forward vs. backward tasks. Furthermore, we explored the relationship between performance on these two WM tasks and auditory speech comprehension. Finally, we examined the complex relationships between WM and TIP to verify whether they are modified by deficient auditory comprehension in PWA.

## Materials and Methods

### Participants

Thirty patients (20 male and 10 female) aged from 27 to 82 years ($\bar{x}$± SD = 59 ± 14 years) participated in the study. They suffered from aphasia after their first stroke (hemorrhage or infarction; lesion age $\bar{x}$± SD = 51 ± 55 weeks). PWA were classified into two subgroups according to time post stroke with a cutoff point at 6 months post onset (Bernhardt et al., 2017): (1) the subacute subgroup (*n* = 14) and (2) the chronic one (*n* = 16). They were recruited in cooperation with neurological and neurorehabilitation clinics located in the area of Warsaw. PWA were right-handed, Polish native speakers with normal or corrected to normal vision. Patients suffered predominantly from disordered auditory comprehension; however, they were able to follow experimental instructions. The hearing level was screened using pure-tone audiometry (Audiometer MA33, MAICO; American National Standard Institute, 2004). The tested frequencies were selected to encompass the frequency spectrum of the presented auditory stimuli, which included 250, 500, 750, 1,000, 1,500, 2,000, and 3,000 Hz.

A description of the patient sample is given in Table 1.

**Table 1:** Characteristics of the patient sample (I, infarction; H, hemorrhage stroke).

**Patients**	**Age range (years)**	**Type of stroke**	**Lesion age (weeks)**
**1**	**26–30**	**H**	**84**
2	26–30	H	19
**3**	**36–40**	**I**	**73**
**4**	**41–45**	**H**	**28**
**5**	**41–45**	**I**	**25**
6	46–50	I	9
7	46–50	I	20
**8**	**46–50**	**I**	**191**
**9**	**51–55**	**H**	**37**
10	51–55	I	12
**11**	**51–55**	**I**	**194**
**12**	**56–60**	**I**	**72**
**13**	**56–60**	**I**	**169**
14	56–60	I	15
**15**	**56–60**	**I**	**67**
16	56–60	I	8
**17**	**56–60**	**I**	**81**
18	56–60	I	12
**19**	**61–65**	**I**	**31**
20	66–70	I	19
**21**	**66–70**	**I**	**57**
22	66–70	I	21
**23**	**71–75**	**I**	**44**
**24**	**71–75**	**I**	**145**
25	71–75	I	6
26	71–75	I	6
**27**	**71–75**	**I**	**47**
28	76–80	I	8
29	76–80	I	24
30	81–85	I	13

*Chronic patients are marked in bold. Both subgroups did not differ significantly in subjects' age and education*.

**Figure 1:**
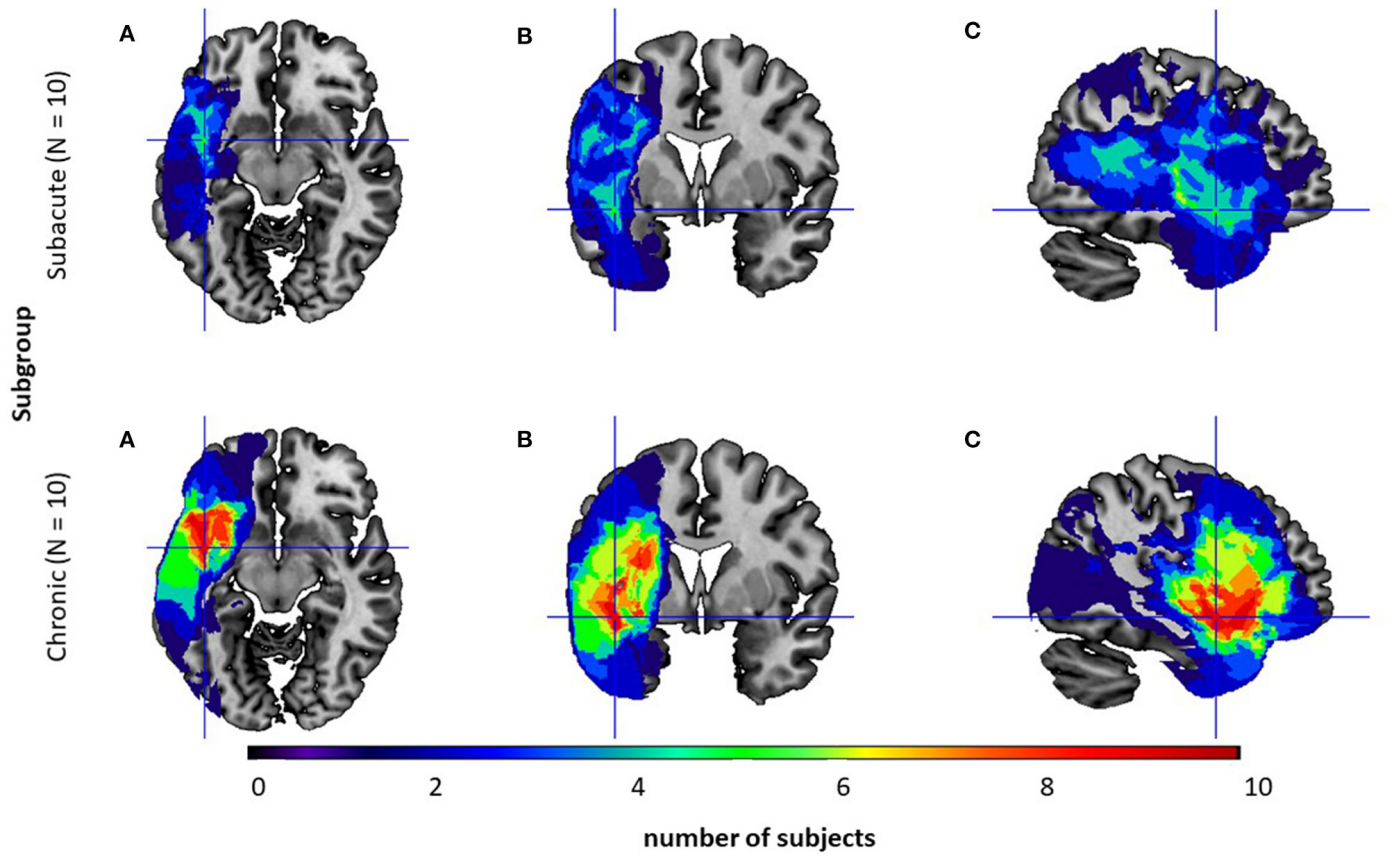
Overlay of the participants' normalized lesion reconstructions on a common brain template in three sections: **(A)** axial, **(B)** coronal, and **(C)** sagittal for two subgroups: the subacute subgroup (top level) and the chronic one (bottom level). Brighter areas (red, orange, yellow, and green) indicate regions of greater overlap, while darker areas (blue and dark blue) indicate lesser overlap.

The location of the lesion was identified by CT or MRI^1^ in 20 (10 subacute/10 chronic PWA) out of the 30 subjects and is visualized in Figure 1.

Neuroanatomical analyses confirmed that subgroups differed in the lesion volume (U = 18.0; *p* = 0.02, Mann–Whitney U test) with significantly greater lesion volume in the chronic subgroup. However, in both subgroups lesions were localized only in the left hemisphere and comprised mainly the following structures: *frontal orbital cortex, middle frontal gyrus, inferior frontal gyrus - pars opercularis and pars triangularis, precentral gyrus, central and frontal operculum, temporal pole, superior and middle temporal gyrus, planum polare, Heschl's gyrus, planum temporale, insular cortex, putamen* with a higher overlap in the chronic subgroup.

The exclusion criteria were: recurrent stroke, global aphasia with poor verbal contact or severe comprehension deficits (which may make the understanding of experimental instructions difficult), visual deficits after stroke, other neurological disorders beside stroke, psychiatric disorders, reported history of head injuries, poor general health, or signs of dementia. These criteria were verified during an interview with the patient's carer or with the clinicians who recruited the patients.

#### Ethical Approval

The study was approved by the Ethical Commission at the University of Social Sciences and Humanities (permission no 26/2017, registered as 35/2017) and was in accordance with the ethical standards of the Helsinki Declaration. All patients provided written informed consent to participate in the study prior to testing.

### Experimental Material and Procedures

The assessment was conducted in a quiet experimental room in the Laboratory of Neuropsychology, Nencki Institute over the course of two or three sessions, depending on each patient's fatigue and health status.

#### Assessment of Auditory Comprehension

Auditory comprehension deficits were assessed on the basis of four language tests: (1) the Token Test, which is a part of the Aachener Aphasia Test (Huber et al., 1983); (2) Comprehension of Syntactic Structures (Smoczyńska et al., 2015); (3) Comprehension of Words (Kertesz, 1982); and (4) Phoneme Discrimination for Pseudowords (Krasowicz-Kupis et al., 2015). In all these tests, the experimenter presented the material verbally to the subjects. Below we summarize briefly the tests used.

(1) The **Token Test** examines auditory comprehension deficits on the sentence level. It is remarkably sensitive to disrupted linguistic processes, e.g., semantic, syntactic, and/or post-interpretative processes. Aphasic patients responded gesturally to verbal commands given by the experimenter. The materials consisted of tokens which differ in color, shape (squares and circles), and size (large and small). The subject followed 50 verbal instructions given in five sections of increasing complexity, e.g., “*Touch the little red circle and the big yellow square.”*
  Outcome measure: percent of correct responses on the entire test (Table 2).
(2) **Comprehension of Syntactic Structures:** participants listened to 16 sentences. During each sentence (e.g., “*The duck is flying above the tree”*) the subject was presented with a set of 4 pictures on a response card indicating 4 different syntactic situations (e.g., above, next to, behind, or under) and was asked to point to the picture corresponding to the situation in the sentence heard.
  Outcome measure: percent of correct responses on the entire test (Table 2).
(3) **Comprehension of Words** consists of 14 trials. In each trial the subject was presented with a set of four different pictures on a single response card. All pictures in each set belonged to the same semantic category, e.g., vegetables. The task was to point to the picture corresponding to the word heard. The first seven test trials consisted of verbs (describing actions; e.g., to crawl) and the next seven trials consisted of nouns (object names; e.g., hole punch).
  Outcome measure: percent of correct responses on the entire test (Table 2).
(4) **Phoneme Discrimination for Pseudowords** consists of 25 pairs of pseudowords in which 18 pairs were different and 7 were the same. The task was to decide whether the pair of pseudowords heard are the same or different and to respond by pointing to one of two response cards (Yes/No). The pseudowords differed in consonants in terms of place of articulation, fricatives, voicing, and nasality.
  Outcome measure: percent of correct responses on the entire test (Table 2).

**Table 2:** Performance on particular language tests (percent of correct responses).

**Patients**	**Token test**	**Comprehension of syntactic structures**	**Comprehension of words**	**Phoneme discrimination for pseudowords**	**Auditory comprehension index (ACI)**
1	88	94	100	96	94
2	–	75	50	64	63
3	22	44	93	68	57
4	72	75	93	76	79
5	20	50	71	76	54
6	92	100	100	100	98
7	98	94	93	92	94
8	62	81	100	76	80
9	84	100	100	96	95
10	90	94	100	96	95
11	70	75	100	100	86
12	40	38	86	92	64
13	52	38	100	100	72
14	70	69	100	96	84
15	–	50	79	100	76
16	82	56	100	72	78
17	54	81	86	84	76
18	96	94	93	100	96
19	54	81	86	64	71
20	–	25	50	48	41
21	66	88	100	96	87
22	16	56	79	92	61
23	18	56	86	52	53
24	–	31	79	52	54
25	94	100	100	88	96
26	68	69	100	72	77
27	62	81	100	96	85
28	–	19	64	44	42
29	82	75	93	100	87
30	42	69	50	84	61

*It was impossible to carry out the Token Test for five patients, as they were not able to follow the introductory session for this test due to specific problems in color differentiation. For these subjects, the auditory comprehension index (ACI) was calculated on the basis of the three remaining tests, which were successfully completed*.

The **Auditory Comprehension Index** (ACI; Table 2) was used as a measure of aphasia severity and defined as the mean percent of correct responses on these four tests.

The **comparison between subacute vs. chronic subgroups** indicated a lack of difference in ACI (t_(28)_ = 0.41; *p* = 0.68; Independent Samples *t* Test).

#### Working Memory (WM)

The efficiency of WM was assessed on the basis of two tests differing in terms of the type of applied material: (1) verbal WM test (VWM developed in our Laboratory) and (2) spatial WM test (SWM; using the Corsi Block-Tapping Test, Vienna Test System; Schuhfried, 2013). In both these tests, sequences of elements (words or squares, respectively) were presented and the subject had to reproduce the order of the presented elements. Each test was conducted in two tasks. In the forward task, the elements were to be reproduced in the same order as they were presented, whereas in the backward task the elements were to be reproduced in the opposite order. Each task becomes more difficult as the number of elements presented in each sequence increases. In both tests, the forward task was conducted first, followed by the backward one.

##### Verbal Working Memory Test (VWM)

Consists of nine concrete unrelated monosyllabic words (in Polish: “*kot, smok, sok, płot, młot, koc, nos, noc, blok,”* in English: “*cat, dragon, juice, fence, hammer, blanket, nose, night, building”*) and a set of nine pictures corresponding to these words.

Before the administration of the test proper, two practice trials were done to ensure that the patient was able to match correctly each of these nine words with the appropriate picture. In each of these two trials, the subject was presented with the nine words separately in a random order and it was verified whether the proper picture had been identified. Performing at least one of these two practice trials correctly was required to proceed with the test proper. Participants who could not manage the practice session were excluded from further testing. The majority of tested subjects were able to correctly match the words with pictures during the first trial.

After successful practice trials, the forward VWM was administered. The experimenter reads a sequence of unrelated words and, after listening to the whole sequence, the subject was asked to reproduce the order of the words by pointing to appropriate pictures. The test starts with a sequence consisting of two words. In subsequent steps, the difficulty was increased by adding one word up to the maximum nine words in the last sequence. In the backward task, the subject was asked to reproduce the words in the opposite order to which they were presented. The backward task proceeded from a sequence of two words to the maximum length of eight words.

There were two trials at each sequence length. The test was terminated when a patient fails to correctly reproduce two sequences of the same length.

Outcome measure: the VWM score^2^ and VWM span for each task of the test.

The *VWM score* was defined as the total number of correctly reproduced sequences in each task. For each sequence reproduced correctly, one point was awarded; the maximum possible scores were 16 (on the forward task) and 14 (on the backward one).

The *VWM span* was defined as the longest sequence length reproduced correctly (at least once); the maximum possible spans were 9 (on the forward task) and 8 (on the backward one).

The **comparison between subacute vs. chronic subgroups** indicated a lack of difference in VWM performance either for scores or spans in the forward and backward tasks (forward score: U = 105 *p* = 0.79; forward span: U = 103.5 *p* = 0.73; backward score: U = 107.5 *p* = 0.85; backward span: U = 109 *p* = 0.92, Mann–Whitney U test).

##### Spatial Working Memory Test (SWM)

The computerized version of the Corsi Block-Tapping Test was used. The subject was shown a constant matrix of nine identical squares located in the same position on the screen in all trials. Two practice trials were initially performed. In each trial, a cursor pointed to two squares on the matrix in a random sequence. After a tone signal, the subject was asked to touch these two squares on the screen in the same order as the cursor. If the response was correct, the subject proceeds to the next practice trial. After correct performance of both practice trials, the proper forward SWM task started with a three-square sequence. The number of squares in the next sequences increased from 3 up to 8. There were three trials for each number of squares. Next, analogous practice trials were performed for the backward task and the subject was asked to recall the sequence of two squares in the opposite of the order that it was presented. After successful backward practice trials, the proper backward task began.

The task was terminated when the subject failed to correctly reproduce the sequence in three consecutive trials.

Outcome measures: the SWM score and SWM span for each task of the test.

The *SWM score* was defined as the total number of correctly reproduced sequences in each task. For each sequence reproduced correctly, one point was awarded; the maximum possible score was 18 (in both forward and backward tasks).

The *SWM span* was defined as the longest sequence length successfully recalled (at least twice); the maximum possible span was 8 (in both forward and backward tasks).

The **comparison between subacute vs. chronic subgroups** indicated a lack of difference in SWM performance either for scores or spans in the forward and backward tasks (forward score: U = 94.5 *p* = 0.47; forward span: U = 105 *p* = 0.79; backward score: U = 110.5 *p* = 0.95; backward span: U = 101 *p* = 0.67, Mann–Whitney U test).

#### Temporal Order Threshold (TOT)

The procedure for measurement of TOT was used in our previous studies and is described in Szymaszek et al. (2009), Szymaszek et al. (2017) and Szelag et al. (2014).

Stimuli were pairs of 1 ms rectangular clicks presented in rapid succession with varied inter-stimulus-intervals (ISI). The paired clicks were presented monaurally, i.e., one click is presented to one ear, followed by a second click to the other ear. The stimuli were delivered at a comfortable listening level through Sennheiser HD 201 headphones. The subject's task was to report the temporal order of clicks within each presented pair—either *right-left* or *left-right*.

The ISIs varied from 1 to 600 ms according to the adaptive maximum-likelihood-based algorithm (Treutwein, 1997). For each trial, ISI was calculated on the basis of correctness achieved in previous responses. This tracking procedure estimated individual TOTs as the minimum ISI between two clicks at which a subject reports their order at 75% correctness. TOT values were assessed using “*Yet Another Adaptive Procedure*” (Mates et al., 2001) on the basis of maximum likelihood parameter estimation. Measurement continued until the TOT value was located with a probability of 95% inside a +5-ms interval around the currently estimated threshold (Treutwein, 1995).

Prior to the task proper, the experimenter familiarized the participants with the TOT task and performed a few practice trials, in which the participants reported the order of two clicks separated by a long interval selected on the basis of our previous studies (e.g., from 600 to 300 ms; see Szelag et al., 2014; Szymaszek et al., 2017). After each response, feedback on correctness achieved was given. During the proper task no feedback on correctness was given.

Outcome measure: TOT value (in milliseconds).

The **comparison between subacute vs. chronic subgroups** indicated a lack of difference in TOT (U = 107 *p* = 0.854; Mann–Whitney U test).

#### Statistical Analyses

To verify the distribution of the resultant data, the Shapiro-Wilk Test was used. Apart from ACI, all variables deviated from the Gaussian distribution: TIP, VWM, and SWM data were skewed positively, with the exception of the backward SWM score, which had a platykurtic distribution.

Therefore, in further analyses, non-parametric statistics were used to investigate:

(1) the differences in performance between the forward and backward tasks of each WM test (VWM/SWM) using the Wilcoxon Signed-Rank Test;

(2) the relationships between the two types of WM (VWM/SWM) and auditory comprehension (indexed by ACI) using Spearman's Rank Correlations, controlling for subjects' Age, Time Post Stroke, and Lesion Volume.

Moreover, Williams-Hotelling test (Williams, 1959) was used to compare coefficients obtained for correlations “VWM-ACI” and “SWM-ACI” for forward and backward tasks.

(3) the relationships between the two types of WM (VWM/SWM) and TIP (indexed by TOT) with Spearman's Rank Correlations, controlling for Age, Time Post Stroke, Lesion Volume, and ACI. Moreover, Williams-Hotelling test (Williams, 1959) was used to compare coefficients obtained for correlations “VWM-TOT” and “SWM-TOT” for forward and backward tasks.

## Results

### Relationships Observed Between VWM and SWM

Individual data for VWM and SWM (scores and spans) on the backward and forward tasks are presented in Table 3 and descriptive statistics are given in Table 4.

**Table 3:** Individual data for VWM and SWM in the backward and forward tasks (scores and spans for each participant), along with temporal order threshold (TOT).

	**VWM**				**SWM**				
**Patient ID**	**Score**		**Span**		**Score**		**Span**		**TOT (in ms)**
	**Forward**	**Backward**	**Forward**	**Backward**	**Forward**	**Backward**	**Forward**	**Backward**	
1	0	0	1	1	8	5	5	3	259
2	5	1	4	2	9	3	5	3	206
3	1	0	2	1	8	4	5	3	202
4	1	2	2	3	9	8	5	5	129
5	1	1	2	2	7	9	4	5	75
6	5	2	4	2	9	8	5	5	136
7	1	0	2	1	5	2	4	3	230
8	2	0	2	1	5	1	4	2	168
9	5	4	4	4	7	7	4	4	113
10	2	2	2	2	5	3	4	3	104
11	5	5	5	4	3	4	3	3	120
12	8	6	6	4	8	10	4	6	97
13	8	6	6	5	9	10	5	5	90
14	2	1	2	2	3	2	3	3	395
15	1	0	2	1	4	3	3	3	134
16	6	3	4	3	5	4	4	3	207
17	5	7	4	5	10	10	5	6	90
18	4	2	4	3	4	5	4	4	257
19	5	6	4	5	5	6	4	4	97
20	2	1	2	2	4	1	4	2	214
21	6	2	5	2	10	10	5	5	76
22	5	5	4	4	5	3	4	3	99
23	4	3	3	3	3	6	3	4	76
24	4	2	3	2	8	7	5	5	67
25	2	1	3	2	4	4	4	4	85
26	10	6	6	4	11	9	6	6	60
27	2	1	2	2	7	6	4	4	100
28	2	1	2	2	9	8	5	4	114
29	8	8	6	6	10	10	5	5	55
30	4	2	3	2	7	4	4	3	101

*A score of 0 reflects situations where subjects did not score any points on the test, even though they successfully passed the practice trials*.

**Table 4:** Descriptive statistics for VWM and SWM on forward and backward tasks: medians (Me) and ranges of obtained scores and spans.

**Test**		**Task**	**Me**	**Range**
VWM	Score	Forward	4	0–10
		Backward	2 ^]***^	0–8
	Span	Forward	3	1–6
		Backward	2 ^]**^	1–6
SWM	Score	Forward	7	3–11
		Backward	5.5 ^]*^	1–10
	Span	Forward	4	3–6
		Backward	4	2–6

Asterisks indicate significant differences in medians between forward and backward tasks for VWM and SWM:

* p < 0.05;

** *p < 0.01*,

*** *p < 0.001*.

For VWM, both the median score and span were significantly higher on the forward than the backward task (*Z* = −3.520; *p* < 0.001 and *Z* = −3.065; *p* = 0.002 for score and span, respectively) indicating better performance on the forward than the backward task. For SWM, the median score on the forward task was also significantly higher than on the backward task (*Z* = −2.363; *p* = 0.018), but the difference in span was nonsignificant (*Z* = −1.906; *p* = 0.057).

**Figure 2:**
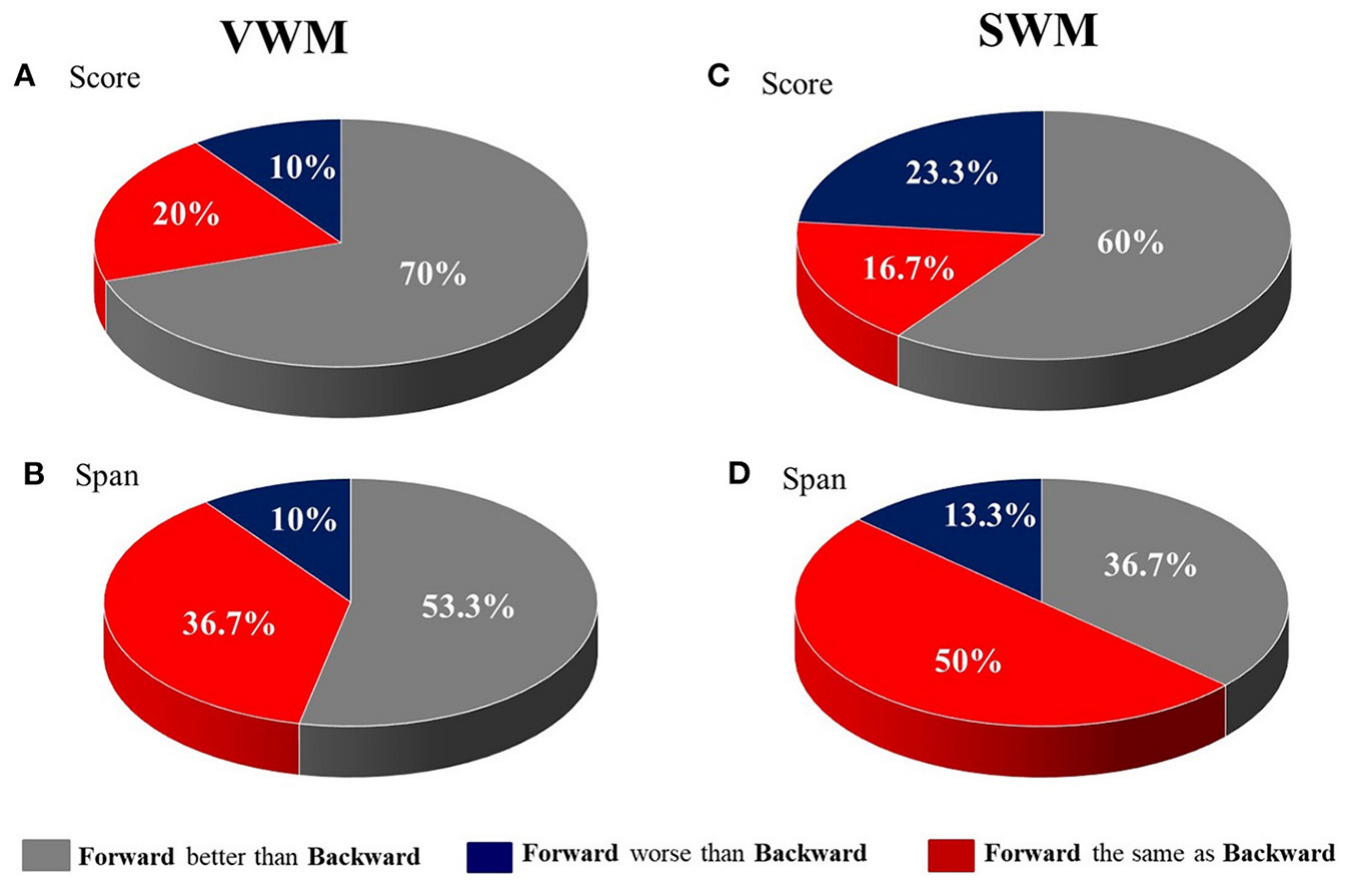
The distribution of patients (in %) with differences in performance between the forward and backward tasks for **(A)** VWM score, **(B)** VWM span, **(C)** SWM score and **(D)** SWM span.

These results are displayed in Figure 2, which shows the percent of patients with a difference in performance between tasks (forward vs. backward) on VWM and SWM. In particular, for scores on both VWM and SWM, as well as for span on VWM, the majority of patients performed better on the forward than on the backward task (Figures 2A–C for VWM and SWM, respectively). In contrast, for SWM span, 50% of subjects had similar performance on the two SWM tasks (Figure 2D).

### “WM – Auditory Comprehension” Relationships

The results of correlational analyses between WM (for both VWM and SWM indexed by scores and spans) and auditory comprehension (reflected in ACI) indicated divergent results for these two types of WM tests (Table 5).

**Table 5:** Spearman's rho correlation coefficients and significance levels between particular VWM and SWM outcome measures on the forward and backward tasks (scores and spans) and auditory comprehension index (ACI).

**Test**			**ACI**	**ACI controlling for Age**	**ACI controlling for Time Post Stroke**	**ACI controlling for Lesion Volume**
VWM	Score	Forward	0.702^***^	0.684^***^	0.709^***^	0.626^**^ (0.664^***^)
		Backward	0.822^***^	0.813^***^	0.831^***^	0.759^***^ (0.774^***^)
	Span	Forward	0.734^***^	0.719^***^	0.744^***^	0.645^**^ (0.679^***^)
		Backward	0.764^***^	0.750^***^	0.767^***^	0.739^***^ (0.767^***^)
SWM	Score	Forward	0.316^***^	0.254	0.290	0.333 (0.343)
		Backward	0.638^***^]**^^	0.620^***^	0.642^***^	0.741^***^ (0.701^***^)
	Span	Forward	0.154	0.090	0.136	0.139 (0.129)
		Backward	0.644^***^]**^^	0.623^***^	0.649^***^	0.786^***^ (0.729^***^)

Asterisks indicate significant correlations:

** *p < 0.01*,

*** *p < 0.001*.

*Square brackets indicate significant differences between coefficients obtained for correlations “VWM-ACI” and “SWM-ACI” for forward and backward tasks (Williams-Hotelling test)*.

*The partial correlations were performed controlling for Age, Time Post Stroke, Lesion Volume (N = 20 PWA, the raw correlation coefficients are given in brackets)*.

**Figure 3:**
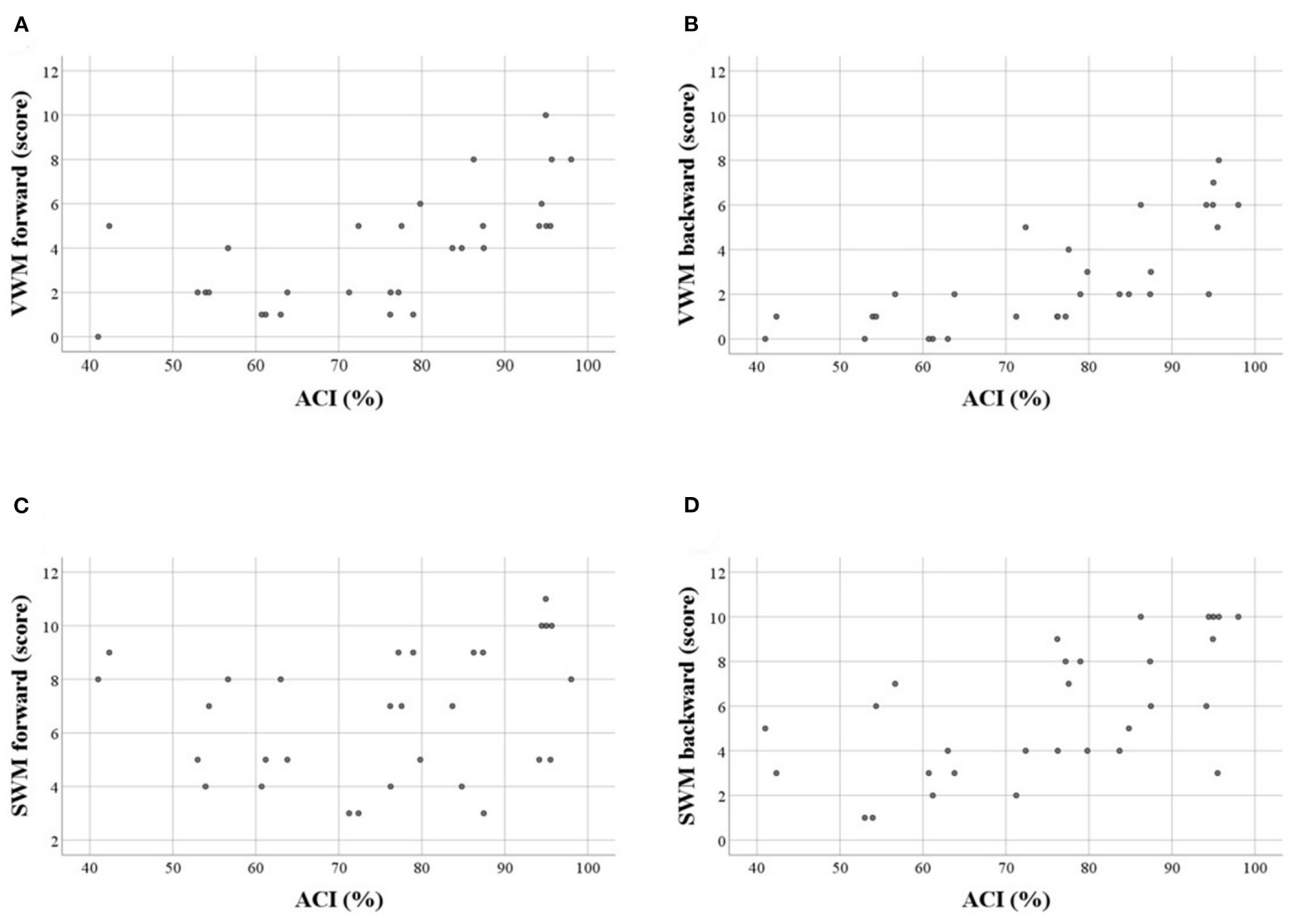
Scatter plots illustrating correlations between WM scores and auditory comprehension index (ACI, in %) in particular tasks: **(A)** VWM forward, **(B)** VWM backward, **(C)** SWM forward, **(D)** SWM backward. Significant correlations were observed for **A,B,D** (see also Table 5). Similar relationships were observed for WM spans.

For VWM, both scores and spans on the forward and backward tasks were strongly positively correlated with ACI. Thus, better VWM performance was accompanied by better comprehension skills. On the other hand, for SWM only the indices of the backward task were correlated positively with ACI, whereas, these correlations on the forward task were nonsignificant (Table 5, Figure 3).

The aforementioned “WM – auditory comprehension” relationships remained relatively stable when the subject's Age was controlled for, indicating a weak influence of Age on the obtained correlations. These “WM – auditory comprehension” relationships were unchanged when controlling for Time Post Stroke and Lesion Volume (Table 5).

### “WM – TIP” Relationships

The pattern of “WM – TIP” correlations differed between the two WM tests, depending on the type of material used (Table 6). In VWM, correlations were mediated significantly by ACI; in SWM, a similar mediation effect was observed on the forward task only.

**Table 6:** Spearman's rho correlation coefficients (and significance levels) between particular outcome measures of VWM and SWM on the forward and backward tasks (scores and spans) and temporal order threshold (TOT) values.

**Test**			**TOT**	**TOT controlling for Age**	**TOT controlling for Time Post Stroke**	**TOT controlling for Lesion Volume**	**TOT controlling for ACI**
VWM	Score	Forward	−0.462^**^	−0.410^*^	−0.462^*^	−0.474^*^ (−0498^*^)	−0.128
		Backward	−0.575^***^	−0.510^**^	−0.575^**^	−0.644^**^ (−0.658^**^)	−0.258
	Span	Forward	−0.483^**^	−0.436^*^	−0.483^**^	−0.502^*^ (−0.524^*^)	−0.140
		Backward	−0.492^**^	−0.444^*^	−0.493^**^	−0.626^**^ (−0.641^**^)	−0.134
SWM	Score	Forward	−0.385^*^	−0.269	−0.402^*^	−0.282 (−0.295)	−0.267
		Backward	−0.661^***^]*^^	−0.580^***^	−0.661^***^	−0.681***(−0.683^***^)	−0.483^**^
	Span	Forward	−0.242	−0.135	−0.247	−0.211 (−0.209)	−0.191
		Backward	−0.689^***^]**^^	−0.619^***^	−0.689^***^	−0.746^***^ (−0.743^***^)	−0.524^**^

Asterisks indicate significant correlations:

* p < 0.05;

** *p < 0.01*,

*** *p < 0.001*.

*Square brackets indicate significant differences between coefficients obtained for correlations “VWM-TOT” and “SWM-TOT” for forward and backward tasks (Williams-Hotelling test)*.

*The partial correlations were performed controlling for Age, Time Post Stroke, ACI, and Lesion Volume (N = 20 PWA, the raw correlation coefficients are given in brackets)*.

In particular, in VWM forward and backward tasks both score and span values correlated negatively with TOT, i.e., better VWM performance corresponded to lower TOT values (more precise TIP). These correlations were relatively stable when the subject's Age was controlled for (indicating a weak contribution of Age to the relationship between VWM and TIP) and became nonsignificant when controlling for ACI. This suggests the relevance of auditory comprehension to the “WM – TIP” relationship in VWM (Table 6).

Different relationships were observed on the backward SWM task, where the significant correlations between score and span values with TOT were independent of auditory comprehension (controlling for ACI, see Table 6). It should be stressed that the mediatory effect of ACI was observed only on the forward SWM task, but not on the SWM backward task, suggesting the crucial role of TIP (but not of ACI) on the backward SWM.

Furthermore, “WM – TIP” relationships remained stable controlling for Time Post Stroke and Lesion Volume indicating a lack of influence of these variables on the obtained correlations (Table 6).

### Summary of Results

1. Performance on forward WM tasks was usually better than on backward ones, with the exception of the SWM spans. The discrepancies between the forward and backward tasks were more pronounced for VWM than SWM (Table 4, Figure 2).
2. The severity of auditory comprehension impairment correlated with performance on VWM (on both tasks), as well as with performance on SWM (but only on the backward task). Thus, better comprehension corresponded to better VWM and better backward SWM performance (Table 5, Figure 3).
3. The “WM – TIP” relationship depended strongly on the type of material used in the WM test. For VWM, significant correlations with TIP were observed on both tasks (forward and backward), while for SWM, significant correlations were found for the backward task. The weak correlation between forward SWM and TIP was noted only for score. Moreover, partial correlation analysis controlling for ACI revealed that correlations between TIP and SWM backward indices remained significant, while those for VWM (both forward and backward) and forward SWM score became nonsignificant. This indicates that TIP alone plays the central role in backward SWM. In contrast, VWM performance is strongly affected by auditory comprehension deficits (Table 6).

## Discussion

### The Relationships Between Forward vs. Backward WM Tasks

In both the verbal and spatial WM tests studied here, the forward tasks were significantly easier, i.e., subjects scored better than on the backward tasks, independently of the type of material used (verbal in VWM or visuo-spatial in SWM, Figure 2). The better performance on the forward VWM task is in-line with previous literature reports regarding PWA (Potagas et al., 2011), as well as healthy elderly people (Kessels et al., 2008). The neural mechanisms underlying this relationship will be discussed subsequently.

It should be stressed that for SWM we observed a smaller discrepancy between the number of subjects who performed better on the forward than the backward task (Figures 2C,D). Although for most participants (60%) the forward task was still easier than the backward one, a substantial number of patients (23.3%) scored better on the backward SWM. This reduced disproportion was more pronounced for span, as 50% of participants performed equally on the forward and backward SWM tasks.

A similar pattern of results for SWM was noticed by Potagas et al. (2011), where PWA performed significantly better on the forward than the backward task (Corsi Block-Tapping Task). On the other hand, in healthy elderly, Kessels et al. (2008) reported different results, i.e., the forward and backward SWM tasks (Corsi Block-Tapping Task) were equally difficult. Such differences in performance between patients and the normal sample may suggest that different processes contribute to the backward SWM in these two subject populations. In the normal sample, both forward and backward tasks rely only on the visuo-spatial sketchpad. In contrast, for PWA (especially backward task) seems more demanding and may engage additional compensatory processes besides those typical for healthy subjects which might be disrupted. Our results indicated that these processes were related to complex interrelations between WM, auditory comprehension, and TIP. These interrelations are discussed in detail below.

### “WM – Auditory Comprehension” Relationships

Our results indicate that, in PWA, comprehension deficits correlate strongly with VWM performance on both forward and backward tasks independently of Age, Time Post Stroke and Lesion Volume. This is in-line with the results of several other studies indicating that VWM requires the storage (maintenance) of memory traces coded verbally in the phonological loop subsystem. In backward VWM, in addition to the maintenance process, there is also a process of manipulation of verbal traces (Baddeley, 2001). The similar magnitude of correlation coefficients (Williams-Hotelling test nonsignificant, Table 5) between ACI and both VWM tasks may suggest that PWA display basic deficits in the maintenance of verbal material in the phonological loop regardless of manipulation of verbal material in the backward task. These basic maintenance deficits (involved in both forward and backward tasks) seem crucial for VWM performance in PWA and reflect the major language problems caused by the brain lesion.

As mentioned in the Introduction, our results regarding associations between VWM performance and language competency are supported by several previous studies. For example, Potagas et al. (2011) tested both subacute and chronic patients within one group or Laures-Gore et al. (2011) examining only subacute patients showed that performance on both forward and backward VWM tasks (Digit Span) is associated with the severity of aphasia (assessed by the Boston Diagnostic Aphasia Examination – Short Form; BDAE-SF; Tsapkini et al., 2010 and the Western Aphasia Battery WAB; Kertesz, 1982). In both these studies, the severity of the language deficits in PWA correlated with the performance on both forward and backward VWM with comparable correlation coefficients. It seems that the phonological loop may be engaged in both VWM tasks and plays a crucial role in the performance thereof. In PWA, distortion to the phonological loop due to the brain lesion impairs the ability to encode incoming auditory verbal input (maintenance), resulting in manipulation being performed on disordered traces during backward VWM.

This supports the thesis that PWA display not only receptive language deficits measured with standard language tests, but also deficient processing of verbal material in inner speech on both forward and backward WM tasks.

In our study, on the other hand, backward SWM performance was also associated with auditory comprehension, while that of the forward SWM task was not (Table 5). These results seemed independent of subject's Age, Time Post Stroke and Lesion Volume. This may suggest that, in the forward task, the presented material was processed using visuo-spatial (non-verbal) cues, regardless of the severity of the patient's comprehension deficits. This is in accordance with the classic view of the Corsi Block-Tapping Task as a nonverbal, visuo-spatial analog of the verbal span tasks. Such visuo-spatial operations seemed to be relatively preserved in PWA, indicating lesions to the left but not right hemisphere (see Figure 1).

The correlation between the performance on the backward SWM task and ACI in our study indicated a contribution of some verbal strategies to SWM, which may be applied during the coding, rehearsing, or recalling of spatial stimuli. However, the present study cannot clarify at which stage such processes are involved and further studies are needed. Other evidence for the engagement of verbal strategies during performance of the backward Corsi Block-Tapping Task was provided by Vandierendonck et al. (2004), who documented worsened performance in a healthy population when verbal strategies were suppressed by an extra articulatory task.

Literature studies did not provide any clear insight into relationships between the forward or backward SWM task and auditory comprehension. For example, Potagas et al. (2011), using the Corsi Block-Tapping Task, reported that performance of both forward and backward SWM correlated at a similar magnitude with language deficits measured using BDAE-SF. On the other hand, in a study by Paulraj et al. (2018), neither forward nor backward spatial span correlated with language abilities measured with the WAB. This disagreement may result from *inter alia* different patient pool tested and indicates that future studies are needed to explain the contribution of language processes to SWM.

Regarding neuroanatomical issues, in our study all patients indicated left-hemispheric lesions, while the right hemisphere was intact (Figure 1). Traditionally, visuo-spatial abilities are associated with right hemispheric processing (De Renzi et al., 1977; Ratcliff, 1979). Nevertheless, some literature studies (Paulraj et al., 2018) have suggested that visuo-spatial deficits can also be observed in patients with lesions in the left hemisphere. This may be explained by the evolutionary hypothesis of Kasselimis et al. (2018), which suggests that cortical areas of the left hemisphere, initially dedicated to visuo-spatial processing, subsequently evolved to support language functions. Therefore, in some cases, left hemispheric areas might still engage in their initial visuo-spatial processes supporting right hemispheric areas. Hence, lesions to the left hemisphere might hinder the processing of visuo-spatial information, especially in more demanding tasks. This hypothesis may help to explain the results observed in our study.

Further support for the involvement of verbal processes in visuo-spatial operations was found by Kasselimis et al. (2013). They reported that patients with left-hemispheric lesions without aphasia performed significantly better on the SWM task than PWA. This may suggest that impairment of the left hemispheric areas dedicated to language selectively worsens SWM performance.

### “WM – TIP” Relationships

To explain the “WM – TIP” relationships, we refer to the functional taxonomy of mental activity proposed by von Steinbüchel and Pöppel (1993; see also Szelag et al., 2011; Nowak et al., 2016) which distinguish two types of cognitive functions: content-related (“what”) functions and logistic (“how”) ones. Content-related functions correspond to the content of our subjective experience, e.g., language, perception (auditory, visual), and memory. On the other hand, the logistic functions constitute the neural base for content-related functions. Accordingly, TIP may be considered to be a logistic function and play a crucial role in WM, as well as in other content-related functions (Szelag et al., 2015, 2018; Jablonska et al., 2020)

Previous reports indicated the co-existence of TIP and language deficits in PWA (von Steinbüchel et al., 1999; Wittmann et al., 2004; Oron et al., 2015; Szelag et al., 2015). On this basis, one may anticipate that VWM is also associated with the efficiency of TIP because of verbal material processing. This may be supported by our results discussed above (Table 6), indicating that, in VWM, the maintenance processes are mainly related to disordered comprehension.

Many previous studies have suggested that TIP creates the neural frame for processing verbal material, including auditory comprehension. Accordingly, our results showed the dominating role of auditory comprehension in VWM, as the “TIP – VWM” correlations became nonsignificant when controlling for ACI (Table 6). Due to massive language impairment, distortion on the level of the phonological loop plays the major role in overall WM performance for verbal material. In such situations, the role of TIP is masked by the language impairment caused by the brain lesion.

In contrast, in the backward SWM, TIP (but not ACI) played the crucial role, as the “backward SWM – TIP” correlation still remained significant when controlling for ACI (Table 6). As discussed above, in PWA the backward SWM may engage some verbal strategies, in addition to the visuo-spatial sketchpad. However, more advanced processes seemed the most important here, i.e., manipulation (which requires the central executive component of WM). Millisecond TIP provides a defined temporal frame for such executive processes.

Support for TIP contributing to manipulation WM processes, but not to maintenance ones, can be found in a study by Jablonska et al. (2020) on healthy elderly people who performed the auditory verbal n-back task. The authors argued that the manipulation in the n-back task involves continuous reorganization and online updating of information. For these dynamic resources, efficient TIP in the millisecond range is crucial.

It seems evident that impaired auditory comprehension may interfere with the performance of verbal tests. However, some authors (Schumacher et al., 2019) based on a detailed behavioral assessment of both verbal and non-verbal performance combined with structural imaging have demonstrated that in PWA the separable behavioral- brain components for attention, executive functions and language features may be identified.

To summarize, we expected that the backward tasks for both VWM and SWM would require manipulation, which is a dynamic process related to precise TIP. But because of the brain lesion causing language impairment, this hypothesis was confirmed only for SWM. In the backward VWM task, due to the massive language impairment, disordered comprehension plays a primary role in PWA, in contrast to the backward SWM task, in which TIP was the crucial factor.

### Limitations of the Current Study and Directions for Future Research

The current study has some limitations. Firstly, due to the correlational design we cannot speculate on causal relationships between WM, auditory comprehension, and TIP. Future experiments involving suppression of particular cognitive functions may be helpful in elucidating causality between the elements of the proposed model.

Some discrepancies between our results and previously published studies involving PWA may be caused by the relatively small samples in these studies, various inclusion criteria, as well as different impairment profiles of PWA. In our study, 30 PWA were included, with relatively high variability of Age (ranging from 27 to 82 years). Nevertheless, the confounding factors were identified and controlled in data analyses.

In our study ACI was considered as the index of aphasia severity, being aware of that it is related selectively to auditory comprehension abilities. However, due to lack of Polish version of any validated and normalized language battery (e.g., the BDAE or AAT) we are not able to provide a global aphasia severity index.

Moreover, the studied sample included PWA in both subacute and chronic phase. Such approach was previously applied by other authors (Potagas et al., 2011; Kasselimis et al., 2018) investigating “language-WM” interrelations. As no group difference in behavioral performance was noted (probably because of a greater lesion volume in the chronic group, Figure 1), correlational analyses were conducted on the whole sample. However, Time Post Stroke (*N* = 30 PWA) and Lesion Volume (*N* = 20 PWA) were controlled for and did not modify the results. It is however possible, that the dynamics of cognitive restitution in both language and memory domains progress differently in the subacute and chronic phase. Therefore, it would more conclusive, in the further studies, to test PWA in the same stroke phase with comparable prognostic factors including neuroanatomical characteristics.

To elucidate the relationships between WM and TIP in the general population, healthy controls should be also tested. This could indicate whether the obtained results are ubiquitous or typical for PWA who display impaired TIP, WM, and language comprehension.

## Data Availability Statement

The raw data supporting the conclusions of this article will be made available by the authors, without undue reservation.

## Ethics Statement

The studies involving human participants were reviewed and approved by the Ethical Commission at the University of Social Sciences and Humanities (permission no 26/2017, registered as 35/2017). Written informed consent to participate in this study was provided by all patients.

## Author Contributions

MC: subject recruitment, acquisition, analysis and interpretation of data, and manuscript writing. ES: interpretation of data and manuscript writing. TW: analysis and graphical presentation of neuroanatomical data. AS: conceptualization and study design, subject recruitment, acquisition, analysis and interpretation of data, and manuscript writing. All authors contributed to the article and approved the submitted version.

## Conflict of Interest

The authors declare that the research was conducted in the absence of any commercial or financial relationships that could be construed as a potential conflict of interest.

## Footnotes

^1^All images were acquired on a 3T MRI scanner (Magnetom Trio TIM VB17) using a 12-channel head matrix coil. Parameters of MRI sequences: T1 MPR isotropic sequence – TR=2530 ms, TE=3.32 ms, TI=1100 ms, FA=7 deg, voxel size=1x1x1 mm, 176 sagital slices, IPAT=2, TA=6:03 min. T2 SPC isotropic sequence – TR=3200 ms, TE=402 ms, voxel size=1x1x1 mm, 176 sagital slices, IPAT=2, TA=4:43 min. DTI isotropic sequence – TR=8300 ms, TE=87 ms, voxel size 2x2x2 mm, 64 axial slices, IPAT=2, TA= 2 x 9:26 min (AP and PA phase encoding direction), b-value1 = 0 s/mm2, b-value2 = 1000 s/mm2. Patients' MRI images were spatially normalized with appropriate templates (T1 and T2 standard MNI templates) using the SPM12 toolbox. Patients' CT images were first filtered to remove bone structures from the image. The images prepared in this way normalized very well to the Proton-density template from the SPM12 toolbox. Lesion areas were pre-segmented by the image intensity thresholding (watershed algorithm in the lesion region) and then manually corrected based on MRI T1, T2 and diffusion weighted images or CT images using the MRICroN software.

^2^As scores are usually more sensitive to a subject's performance, we concentrated mainly on this indicator.
